# Supplementary material for: Assessing structure-function impacts on Vitellogenin by leveraging allelic variant found in honey bee subspecies Apis mellifera mellifera
Source: iScience. 2025 Jul 29;28(9):113241. doi: 10.1016/j.isci.2025.113241 (PMC12496180; doi:10.1016/j.isci.2025.113241)
Supplement: Document S1. Figures S1–S7, Table S1, and Method S1 [file mmc1.pdf]

iScience, Volume *28*

Supplemental information

Assessing structure-function impacts on Vitellogenin by leveraging allelic variant found in honey bee subspecies *Apis mellifera mellifera*

Vilde Leipart, Oriol Gracia Carmona, Christine Orengo, Franca Fraternali, and Gro V. Amdam

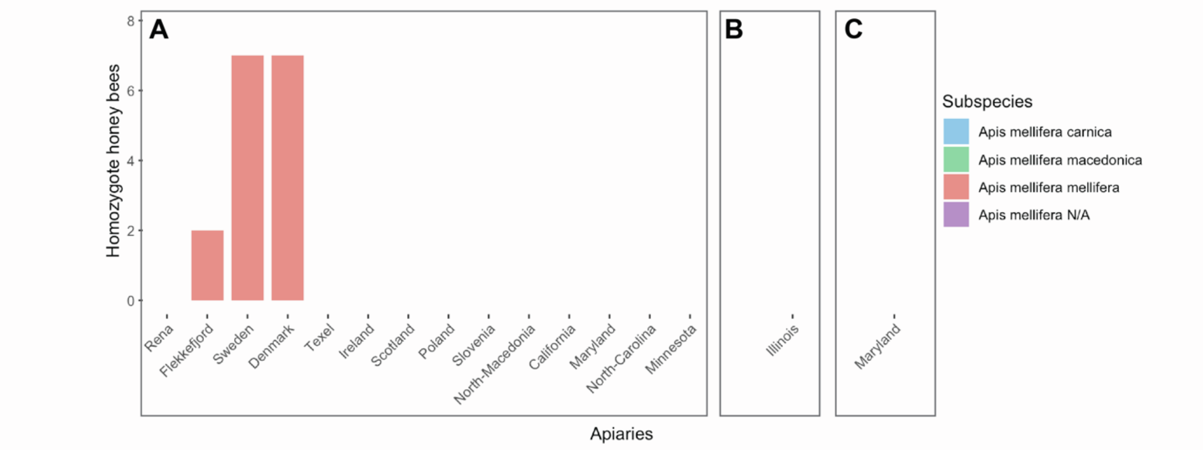

***Figure S1 Homozygotic honey bees: A** The identified homozygotic bees for p.N153_V155del. The number of honey bees (y-axis) is plotted per apiary (x-axis). The apiaries are colored by subspecies (legend). **B-C** No homozygotic bees were identified for p.S844_V845del (B) or p.R1669 (C).*

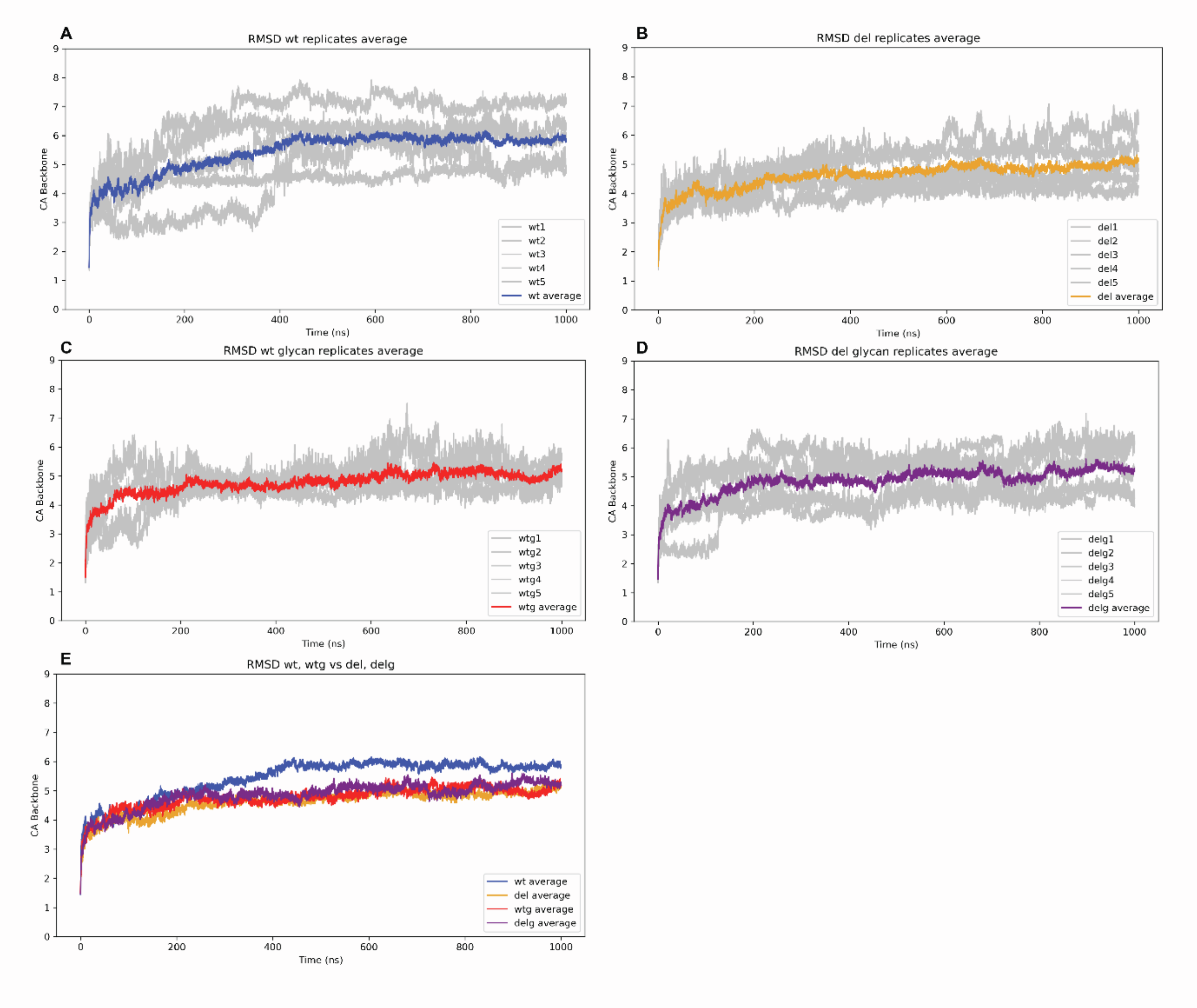

***Figure S2 RMSD after 1µs for all systems.** The RMSD between the Cα atoms (y-axis) over time (x-axis) for each replicate are plotted in grey, while the mean RMSD is plotted in colors. **A** wt replicates and mean (blue). **B** del replicates and mean (orange). **C** wtg replicates and mean (red). **D** delg replicates and mean (purple). **E** Plott of all mean RMSD values from all systems.*

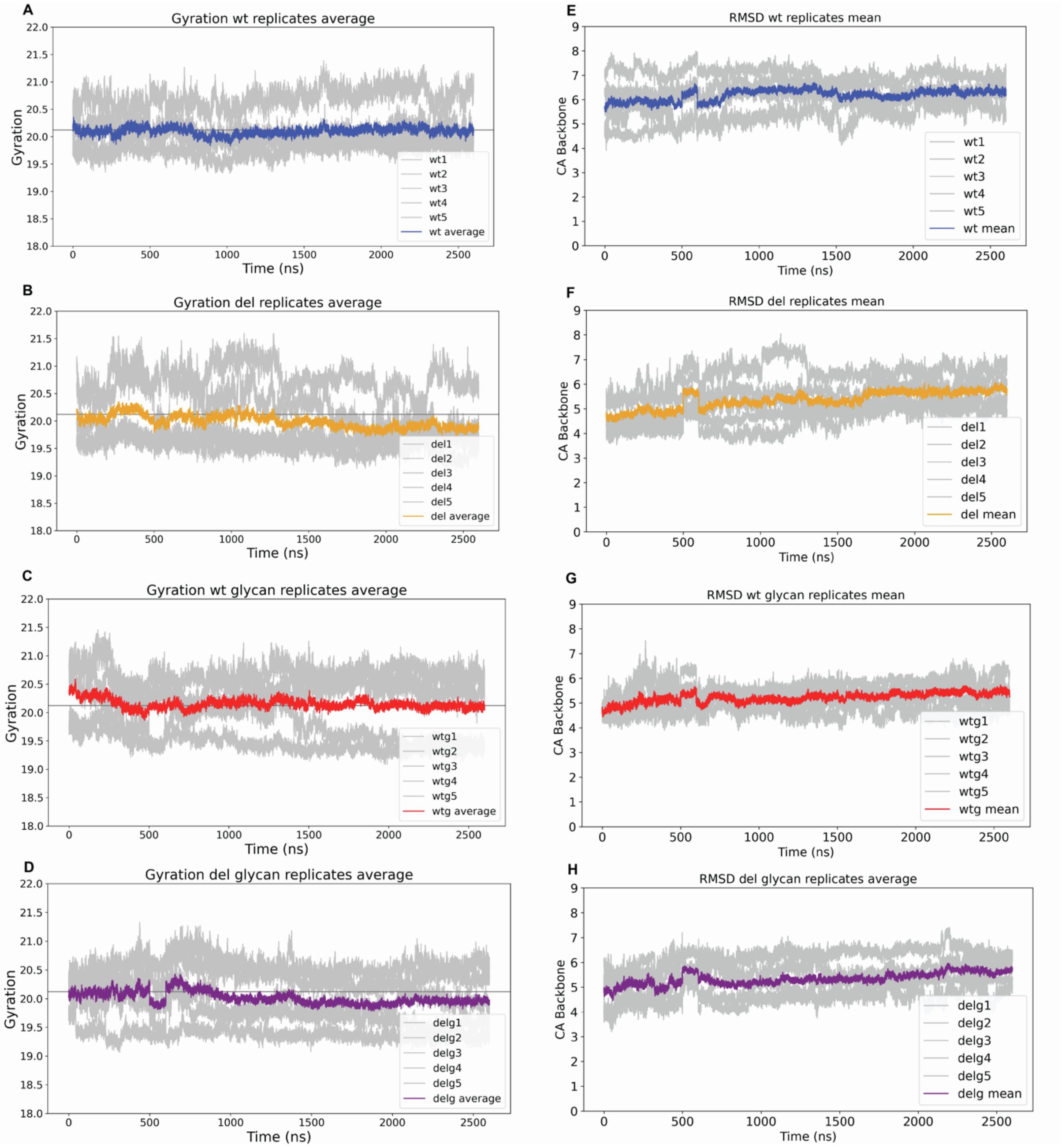

Figure S3 Rg and RMSD after 3μs for all replicates per system: A-D: *The Rg (y-axis) over time (x-axis) for each replicate are plotted in grey, while the mean Rg is plotted in colors. The grey horizontal line shows the Rg of the starting wt model (20.12 Å).* ***E-H*** *The RMSD for the Cα atoms (y-axis) over time (x-axis) for each replicate are plotted in grey, while the mean RMSD is plotted in colors.* ***A and E*** *wt replicates and mean (blue).* ***B and F*** *del replicates and mean (orange).* ***C and G*** *wtg replicates and mean (red).* ***D and H*** *delg replicates and mean (purple).*

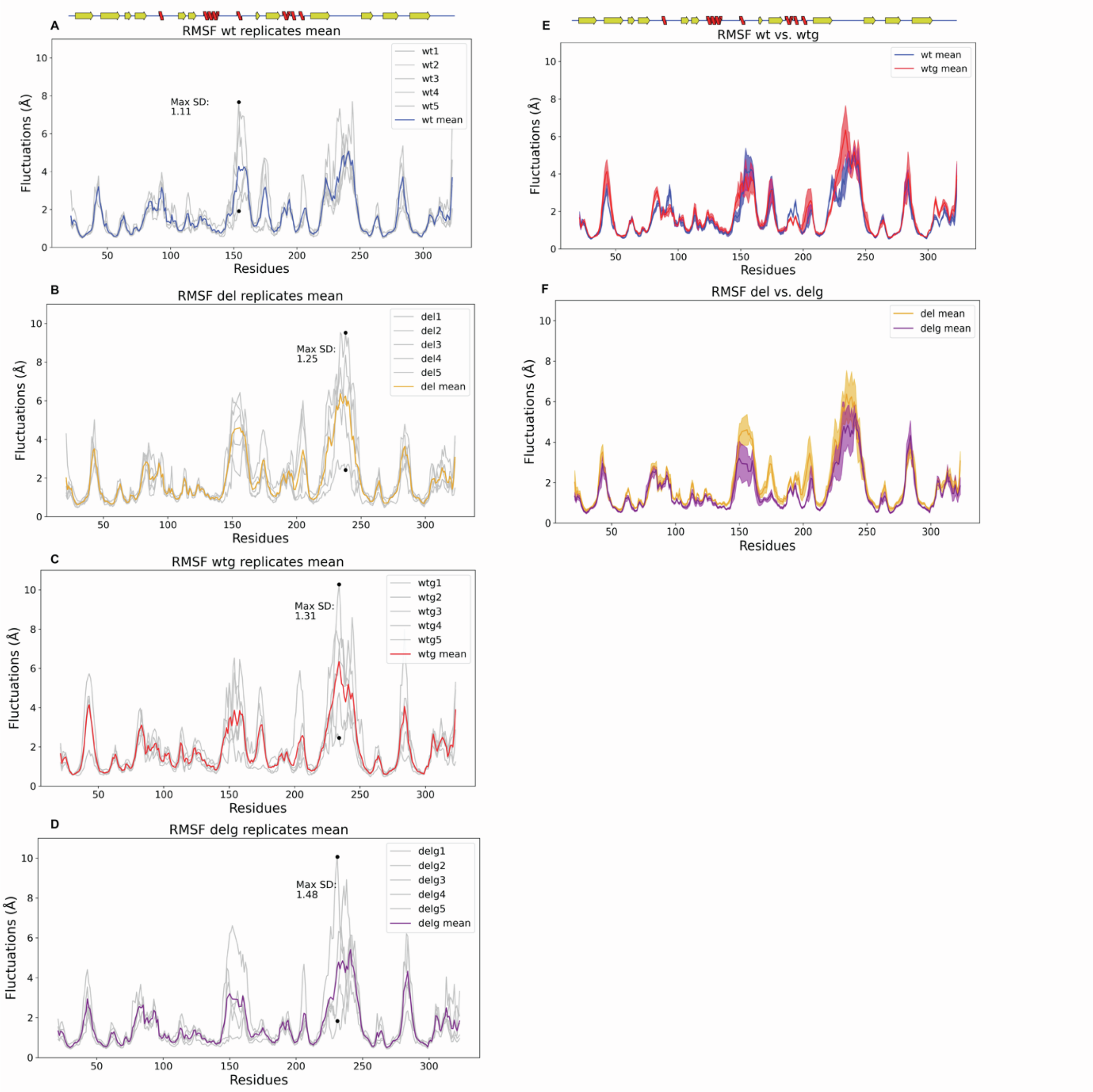

Figure S4 RMSF after 3µs for all replicates per system and analysis: A-D: *The RMSF (y-axis) for the Cα atoms (x-axis) for each replicate are plotted as separate grey lines, while the mean RMSF is plotted in colors. The maximum SD observed between the replicates is plotted as black dots.* ***A*** *wt replicates and mean (blue).* ***B*** *del replicates and mean (orange).* ***C*** *wtg replicates and mean (red).* ***D*** *delg replicates and mean (purple).* ***E*** *The mean RMSF (y-axis) for each Cα in the wt (blue) and wtg (red) systems (x-axis, aa 21-323). The mean values are plotted as a solid line, while the ± SD values are transparent. On top of the plot is the 2D representation of the β-barrel secondary structure elements, the same as in Fig 1A.* ***G*** *has the same plot as in panel F, but the mean RMSF values are from the del (orange) and delg (purple) systems.*

1

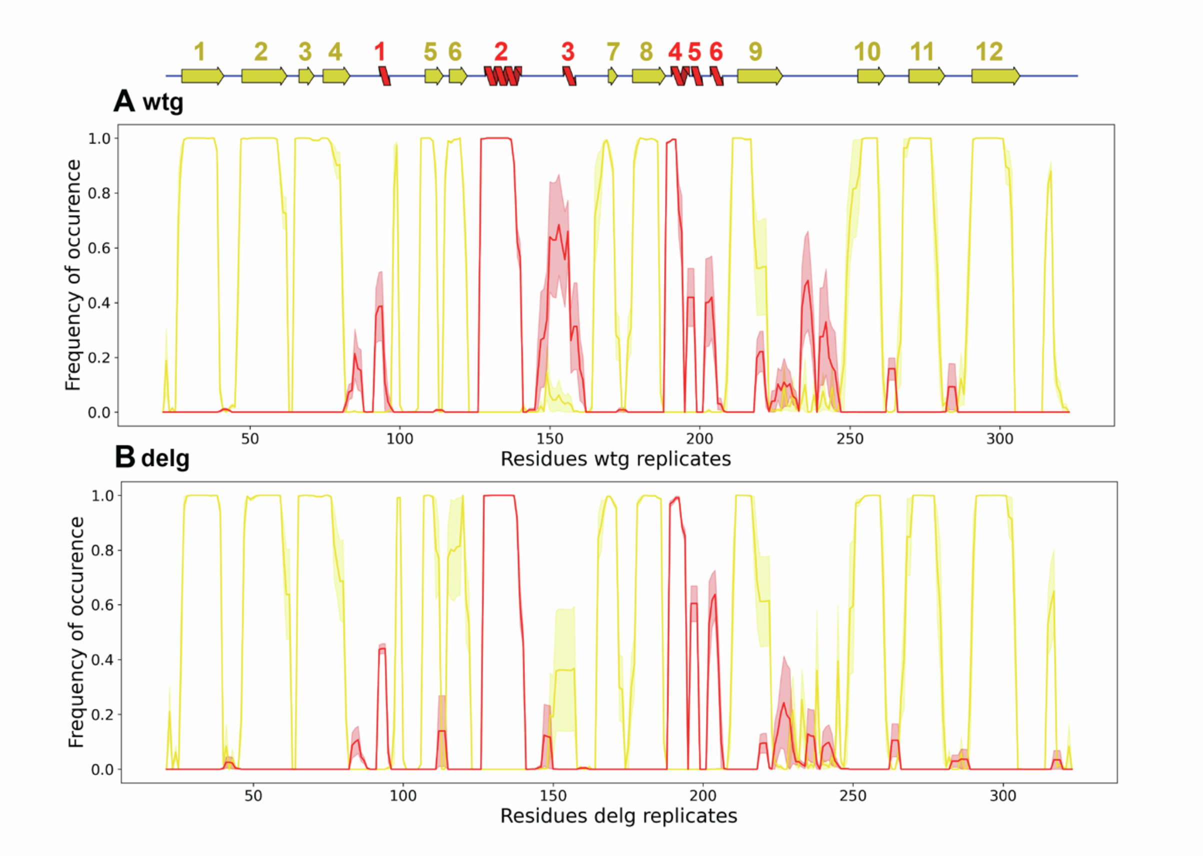

***Figure S5 Secondary structure element assignment for the glycosylated systems. A** The mean frequency of β-strands or α-helices (y-axis) are plotted per Cα (x-axis) of the wtg system. The mean frequency is plotted as a solid line, while the ± SD values are transparent. On top of the plot is the 2D representation of the β-barrel secondary structure elements (β-sheets in yellow and α-helices in red). **B** is the same plot as in panel A, but the mean frequency and SD values are from the delg system.*

2

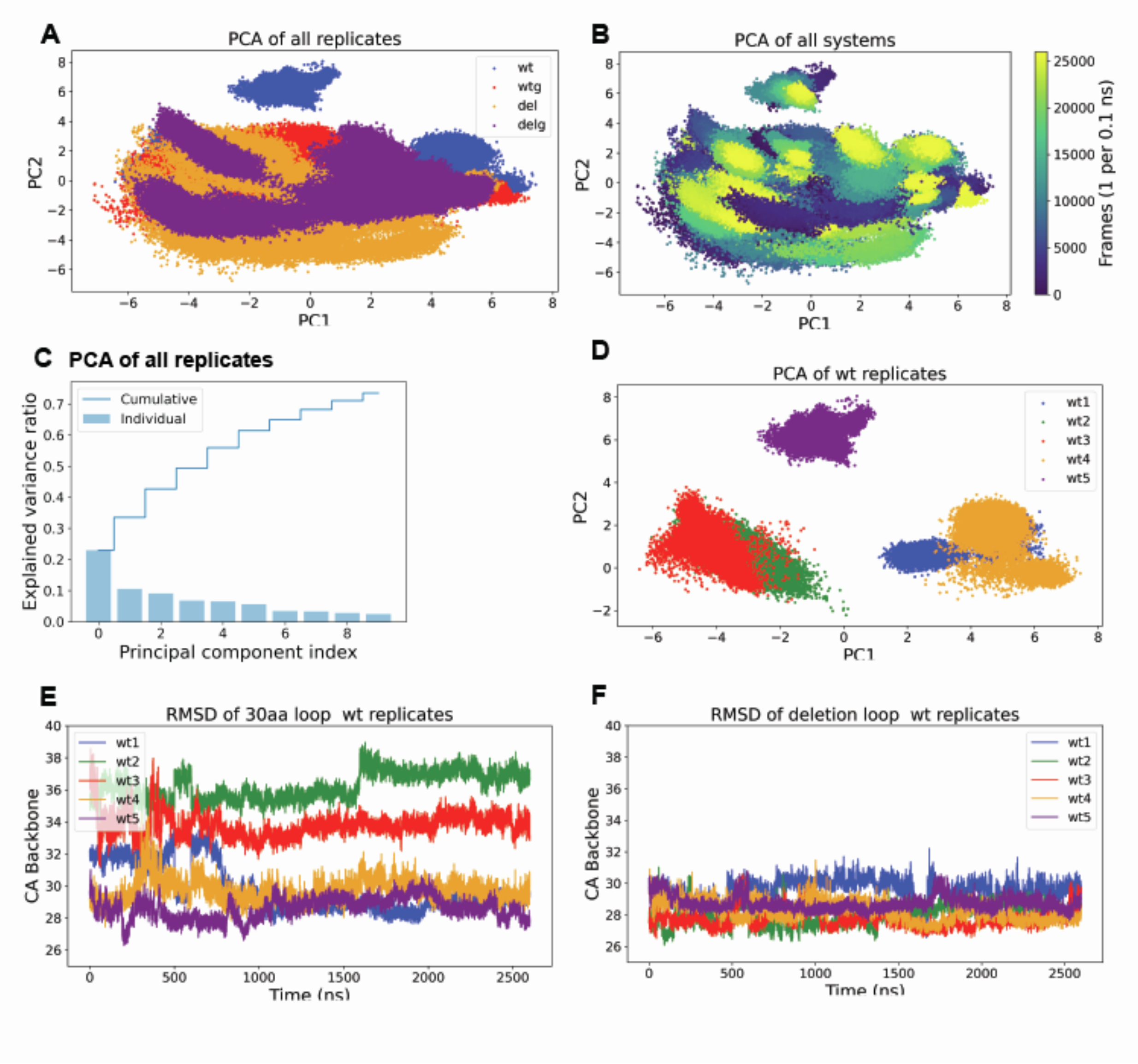

***Figure S6 PCA analysis for all systems: A** PCA plot of two dimensions (x-axis: PC1, y-axis: PC2) of all 20 replicates, wt: blue, del: orange, wtg: red, delg: purple. **B** Same plot as in panel A, but colored after frames (1 frame per 0.1 ns). **C** The explained variance (x-axis) by PCA for 10 dimensions (y-axis). **D** Same plot as in panel A, but only includes the wt replicates: 1 (blue), 2 (green), 3 (red), 4 (orange), and 5 (purple). **E** The RMSD for the Cα atoms of the 30aa-long loop when (y-axis) over time (x-axis) for each wt replicate with the same colors as in panel D. **F** Same plot as in panel E, but the mean RMSD for the deletion loop.*

3

```
                                               141                           170
Q868N5_VIT_APIME/20-323/OS=Apis               QVDT--QGENAV----KVNSVQVPTDD-------EPYASFKAME
Q17083_ATHRO/19-322/OS=Athalia                QVDT--QGENAM----KSRYNQEPEGD-------QAYATYKAME
O17428_9HYME/20-324/OS=Pimpla                 QVDT--QGENLM----ASKYNQEPEDE-------GVTAMFKTME
B2BD67_9HYME/21-324/OS=Pteromalus             QVDT--QGENAI----RSKHNQFPEGK-------QPYALFKVME
Q698K6_ENCFO/22-326/OS=Encarsia               QVDA--RGQNAE----YSKHNQLPEGK-------QPYAIFKTRE
C4PB33_BOMIG/20-323/OS=Bombus                 QVDS--LGENAI----RTSEMQIPTDE-------HPYGMFRAME
C7F9J8_BOMHP/20-323/OS=Bombus                 QVDS--LGENAI----RTSETQIPTDE-------YPYGMFRAME
Q7Z1M0_VIT1_SOLIN/17-310/OS=Solenopsis        QVDL--QGENVI----ASSDNQIPDDS-------QPFGVYKAME
Q2VQM6_VIT2_SOLIN/22-327/OS=Solenopsis        QVDT--QGENAI----NSKSIQVPSDE-------SFAATFKAME
O02024_RIPCL/18-323/OS=Riptortus              QVDT--QGKNLK----DSKINLVPSGQ-----SGEPMGVYKTME
Q0ZUC7_HOMVI/20-323/OS=Homalodisca            QVDT--QAENLQ----KSRINSLPTQE-------TVNGVYKTME
Q05808_VIT_ANTGR/21-324/OS=Anthonomus         QMDL--QGENAL----QNPTSSFPTNE-------YMDAVFKTME
B1B5Z4_9HEMI/26-329/OS=Lethocerus             QIDT--AARNLQ----KSRINQLPAMH-------KPMGVYKTME
Q16927_VIT1_AEDAE/114-419/OS=Aedes            QVDT--RGANLM----HSSKPIHPSKN-------EWNGHYKVME
A7BK94_NILLU/22-336/OS=Nilaparvata            QVDV--TGQNAI----KSRRNIVPNGQ-------QVSGSFKVME
Q9U5F1_9HEMI/31-345/OS=Graptopsaltria         QFDT--EGENIQ----KSRSNSLFDNS------DDPVVTFKTME
Q9GUX5_ANTPE/28-319/OS=Antheraea              QLDL--STHRNI----HGSQDSYDRE--------QKQGLFRKME
Q59IU3_9NEOP/27-318/OS=Saturnia               QLDL--STHRNI----QGSQNRYDQE--------QKQGLFRKME
Q9U8M0_VIT1_PERAM/18-316/OS=Periplaneta       QVNT--QADNLV----RHRYNILPNAS-------TDSAVYSIRE
O76823_BLAGE/21-303/OS=Blattella              QVVV----------------------------DEDKKVYRVFE
Q5TLA5_RHYMA/32-311/OS=Rhyparobia             QLTT--QP--------------------------SFKPVYRVKE
A0A6J0C3D1_NEOLC/30-333/OS=Neodiprion         QVDV--QGENQM----DSTYNQEPEGG-------KAYATFKVME
A0A0S2EH87_GEOPA/19-324/OS=Geocoris           QVDT--QGENLK----KSKINLLPKDQ-----HKGKMAVFKVME
A0A516SN07_ORISA/18-321/OS=Orius              QVDT--QGENLK----KSKINQVPSKG-------QAMGVYKTME
A0A650FKV7_9HYME/21-324/OS=Tetrastichus       QVDT--QGENAE----KSKHNQLPEGK-------QPYAVYKTME
A0A0J7KVJ8_LASNI/25-343/OS=Lasius             QVDT--QGENSI----SSSSNQIPDDE------EQPFASYKVME
A0A7T1IPX1_9HYME/20-323/OS=Centris            QVDS--QGENVI----KSKNTQVPSDS-------DPYGSFVVME
A0A076KX93_9HYME/24-339/OS=Formica            QVDT--QGENRI----KSRSDQIPDNE------QQPFASYKVIE
A0A097QQV0_9HYME/20-329/OS=Osmia              QVDT--QGENIIKDLVKDPSIQIPDDN-------EPYGSFKSME
A0A026WAJ5_OOCBI/22-331/OS=Ooceraea           QVDV--QGENAI----LTKYTQIPSDE-------QPYATFTAME
A0A6B9MT39_SOGFU/22-331/OS=Sogatella          QVDV--TGQNVI----KSRRNIVPNSN-------QVSGSFKAME
M9TDC6_LAOST/22-334/OS=Laodelphax             QVDV--TGQNAI----KSRRNILPQSDS----NQQVSGSFKAME
E2ANT2_CAMFO/30-338/OS=Camponotus             QVDL--SGQNII----PSKYNQMPEDA------QQPFSSYKVME
A0A7R8Z5D1_HERIL/20-322/OS=Hermetia           QVDI--RGQNLM----PSPYNQLPNVQE----NDIPSGLYEVME
V9HZ11_BEMTA/18-321/OS=Bemisia                QVDT--QGQNLK----KSSHNQLPKEN-------KPYGVYKTME
A0A140KFN5_9HEMI/19-303/OS=Glaucias           QVDV--QGQNLK----KSKINDVPKDN-------QHTGIYKTME
A0A126CG82_9NEOP/22-324/OS=Chrysopa           QVDM--QGQNLL----SSRYNQKPEMN-------ENNGNYKTME
Q9U5D6_PLAST/20-324/OS=Plautia                QVDT--QGVNLK----QSKLNNVPQGG-------QQNGVYKTYE
A0A6M2DSL4_9NEOP/1-271/OS=Xenopsylla          QVDT--QGKNLI----KSKYNQLPEGE-------QVNSFFKTME
A0A154NWB0_DUFNO/20-323/OS=Dufourea           QVDT--QGDNAI----RTRSMQVPNDD-------QPYGMFLAME
A0A0N0BC62_9HYME/10-311/OS=Melipona           QVDT--KGEDAF----MTNDKQLPTEE-------LPYGNFRKME
A0A6V7HH51_9HYME/9-312/OS=Heterotrigona       QVDT--KGEDAL----KTNDLQIPTEE-------LPYGNFRKME
E0VZ54_PEDHC/18-321/OS=Pediculus              QVDT--QGVKAI----SSKLNSIPTGD-------ENTAVYKVME
A0A2H5BER7_9HYME/28-328/OS=Polyrhachis        QVDT--GGKRII----PSRYNQVSEDY------YQPYAAYKVME
A0A0A9YCB7_LYGHE/22-326/OS=Lygus              QMDT--TGENLK----KSSYNQIPQAN-------DLTAAFKTTE
A0A158NZ82_ATTCE/23-334/OS=Atta               QIDT--QGENAI----KTRSAQIPVDE-------NSPVIFTAVE
A0A0C9QY44_9HYME/25-328/OS=Fopius             QIDT--QGENMR----SSVSNQPPSDK-------IINAMFKTME
A0A6J1RF71_9HYME/46-346/OS=Temnothorax        QIDI--RGENAI----GGRGTQEPDDN-------QPFGTFKVME
A0A6L2PZZ8_COPFO/1-298/OS=Coptotermes         QVDY--RALKTSN---KGKKN-------------QINTTYKVME
A0A232ETW9_9HYME/20-324/OS=Trichomalopsis     QIDT--SGENVK----KSKRNQLPEGN-------QPFAFFKAME
G8IIT0_VESVU/23-326/OS=Vespula                QADT--QGENLK----GNKNTQIPEDD-------NPFATFRVME
A0A0S2C3U3_RHYFE/19-324/OS=Rhynchophorus      QLDV--RGKNAI----DSPINDLPSED-------KLDGVFKTME
```

```
                                               141                           170
D7P6M3_CULTA/51-356/OS=Culex                  QLDL--FGANLI----KSKYNQLPENE-------TANAVFKTME
T1DDZ0_9DIPT/42-349/OS=Psorophora             QLDL--TGANLI----KSKYNQLPHNE-------SISAVYKTME
A0A336LYD9_CULSO/18-323/OS=Culicoides         QLDL--EGDNEI----ECDKNQFPEKH-------DWTGTFKVME
A0A1W4WFI7_AGRPL/18-324/OS=Agrilus            QLDT--LGQNVI----ESRLNQLPKEG-------SDFAVFKTME
A0A650D4Y5_SCHGR/21-318/OS=Schistocerca       QLDV--RGSNRV----SR-----PVPG-------GDSDIYWTME
A0A5N4AUL5_PHOPY/23-325/OS=Photinus           QLDI--QGVNLM----KDRINQLPEND-------SYSGVYKTME
A0A653CDT2_CALMS/21-316/OS=Callosobruchus     QLDL--KGSRRI----DSEMGS--SET-------GIPSVFKTME
U4UYR7_DENPD/20-323/OS=Dendroctonus           QLDL--QGKRAV----ESHATTLPNDE-------KMEAVFQTME
A0A182YRT3_ANOST/39-344/OS=Anopheles          QLDT--QGAYVI----KSEFNQFPENN-------TLTGVYKTME
A0A224XAS6_9HEMI/18-321/OS=Panstrongylus      QVDT--QAENLK----KSRVNQVPVEG-------QTIGVYKTVE
A0A3Q0JK51_DIACI/82-388/OS=Diaphorina         QVDT--RAENEV----SSRLNQKPKNG-------KPFGTFKTME
A0A1B6CLY7_9HEMI/22-325/OS=Clastoptera        QVDT--QARKLI----KSRLNALPERN-------GMVGMFKTME
A0A1J1I180_9DIPT/77-415/OS=Clunio             QVDT--QAQYLI----KCSSNQLPEPK-------TVNGVFKTME
A7XS62_SPOLT/26-317/OS=Spodoptera             QVDL--STHSST----NNREDYLDRE--------REQGLFKKME
A0A059VBJ4_CORCP/25-316/OS=Corcyra            QVDL--STHRHI----RSSHHNYDKD--------TKQGLFKKME
A0A212ES36_DANPL/27-318/OS=Danaus             QVDL--TSHRNV----KSSHDTYDTQ--------VQQGLFRKME
A0A6J1N8X6_BICAN/30-321/OS=Bicyclus           QVDL--STYRNV----HSSHDTYDKN--------IRQGLFRKME
G3LSH9_CNAME/29-320/OS=Cnaphalocrocis         QVDL--STYRHV----RRQENGFDQQ--------TQQGMFKKME
A0A0S2C3S6_CADCA/28-318/OS=Cadra              QVDL--STNRVM----HNVHDQFDRE--------TYQGQFSKME
Q25269_LYMDI/28-318/OS=Lymantria              QVDL--TNYRLV----NRSQDSYDKN--------SQQGLFSKFE
A0A6M8PV56_PLUXY/37-326/OS=Plutella           QVDL--TPYRTV----RDSHSFFNKE--------TFQGQFYKKE
A0A3G1S403_9NEOP/38-326/OS=Conopomorpha       QVDL--TPYRKA----LGSHDYYDQE--------RSQGQFRKME
A0A7E5VQX3_TRINI/24-302/OS=Trichoplusia       QVDL--STYGHV----HNFPNSYDKE--------SFQGLFKKNE
A8CMX8_HELZE/24-314/OS=Helicoverpa            QVDL--SPYGHV----DNFPNSYDKE--------SFQGIFKKVE
A0A2A4J2Z7_HELVI/24-314/OS=Heliothis          QVDL--STVGHV----HSFPNSYDKE--------TFQGLFKKIE
A0A2S1XV30_THIPU/29-302/OS=Thitarodes         QVDL--SDKHLI----KSAQNYYNER--------AASGSFRKME
A0A649ZV53_ZOPAT/20-320/OS=Zophobas           QLDT--RATNVI----RSSINMLSQDY-------SNSAVYKTME
Q64EZ1_TENMO/20-321/OS=Tenebrio               QLDT--KASNLM----VSSINILTQDD-------SNTAVFKTTE
A0A482WDD5_9CUCU/20-321/OS=Asbolus            QLDT--GAKNSI----PSHYNILSQEG-------SNTAVFKTME
D6W7J2_TRICA/20-321/OS=Tribolium              QLDT--NAENLI----PSSINILTQEG-------SSTAVFKTME
A0A7T7P1J3_9CUCU/21-326/OS=Galeruca           QLDT--KAQNLI----KSQLNVVPKEG-------SNNAVFKTLE
A0A1X9IT98_HARAX/21-318/OS=Harmonia           QLDT--RAENVK----YSEYNILPKEG-------SNNAVYKTVE
A0A7R9IMP2_9NEOP/1-258/OS=Timema              QLDL--TNRNAG----ETADSNVDHKRGRDRLASVADPVYKVTE
A0A5E4R8I7_9NEOP/29-319/OS=Leptidea           QIDL--SPHRNI----HSPRDTYDSA--------IQQGQFRKME
A0A821V7J1_9NEOP/29-322/OS=Pieris             QIDL--STYRNI----NSPGDNYDND--------AKQGLFRKME
E2C964_HARSA/21-297/OS=Harpegnathos           QLDL--SQVQLQ----------TPMKK-----HA-----FVTTE
A0A151JCC5_9HYME/18-300/OS=Trachymyrmex       QLDL--AHIQMQ----------TPLKP-----HG-----FITHE
A0A0U4VTE6_9NEOP/34-334/OS=Reticulitermes     QVNT--QGARII----KDRENNLPQAN-------STNAVFRAME
A0A348G624_ODOMO/19-314/OS=Odontomachus       QFEIDLRTQKKI---------TKPGDS-------HDYGVYTTKE
A0A6P3WQQ6_DINQU/43-341/OS=Dinoponera         QYSE--DLELRS----PQELIVIPKMP-----TKNDYVTYTVRE
A0A2J7R0V8_9NEOP/1-309/OS=Cryptotermes        QLNT--QAVHLI----THRQNHIPGPN-------SDSALYSVME
A0A067QSQ5_ZOONE/19-317/OS=Zootermopsis       QLNT--HAVDVV----KHRQNNVPGPD-------SKSAQFHVIE
A0A182BF83_9CUCU/20-318/OS=Colaphellus        QLNT--SA---------AKLNSLSENQ-------QESAVFRIME
A0A0K0Q025_9CUCU/20-315/OS=Octodonta          QLKI-------------NERSESPE---------EHATHFQVVE
A0A516KLL5_9CUCU/20-313/OS=Agasicles          QLNI--YQGD------------LQKGN-------QNSAKFQVYE
A0A6P8YSF5_THRPL/27-328/OS=Thrips             QIQA------------GSSGNSLHGKE-----QHSEQGVYRVLE
A0A1B6KR29_9HEMI/24-320/OS=Graphocephala      LTDQ--SQERNF----GPNSNKMALS--------QEKDVYHVLE
A0A834XW43_9HYME/26-314/OS=Aphidius           NYGY----------------NDFVKNV-------ETTYNITKLE
A0A7M7GEB3_NASVI/18-329/OS=Nasonia            NIGA--NIAKKR-------------------------EPEFRHME
A0A6I9VWI2_9HYME/28-322/OS=Pogonomyrmex       NIGF--ETQERR--------------------------DRFTIME
A0A411G848_9HYME/23-310/OS=Cotesia            TIGT--DLSDTM-------------------------KSTYRSFE
```

Figure S7 Sequence alignment of insect specific loop: *We repeated the alignment from Havukainen et. al 2011 with 21 insect sequences (Names labelled in bold text). The names also include the UniProtID, the sequence numbering for each protein, as well as the genus. We added 82 additional insect sequences (sequence representatives from class Insecta (Taxon ID: 50557)) from the alignment generated in our study (not bolded names, first and second column). We colored the alignment using the same scheme as Havukainen et. al: Highly conserved residues are labeled in orange, conserved glycines and prolines in blue, and conserved charged residues in red (D, E, R, K, H). In addition, we also find conserved aromatic residues (phenylalanine and tyrosine), shown in pink, and polar residues (asparagine, serine, and glutamate), shown in green.*

Replicates		Deletion loop (aa 141-167)	30aa-long loop (aa 219-249)
wt1	Overall mean	29.54	29.73
	Range (Å)	26.55-32.21	27.58-33.86
	SD	0.72	1.45
wt2	Overall mean	28.03	36.23
	Range (Å)	26.09-31.03	33.85-38.96
	SD	0.64	0.85
wt3	Overall mean	27.74	33.82
	Range (Å)	26.51-30.61	31.17-38.58
	SD	0.56	0.75
wt4	Overall mean	28.25	29.74
	Range (Å)	26.81-31.48	27.44-34.62
	SD	0.60	0.72
wt5	Overall mean	28.74	28.61
	Range (Å)	27.60-30.79	26.28-31.02
	SD	0.45	0.68
wt1-5	Overall mean	28.46	31.62
	Range (Å)	27.42-29.56	30.52-33.46
	SD	0.25	0.34

Table S1 RMSD calculations for the deletion loop and the 30aa-long loop after superimposing the full-length fold. *Rows 1-5 show the calculations for RMSD for each wt replicate. For the two loop regions (columns 3 and 4), we calculate the overall mean for all* $C\alpha$*, the range of difference in Å, and the standard error of the mean (SD). The last row includes the same calculations for RMSD, but we are comparing all the wt replicates using the mean values*

Method S1: Molecular dynamics simulations

The PDB files for each system were uploaded to generate a PDB in CHARMM-GUI [1–3]. The waterbox size was selected by default (Rectangular with an edge distance of 10.0 Å). Ions were added using the default options (Monte-Carlo, KCl) to solvate the molecule. The grid information was generated automatically using the Particle Mesh Ewald Fast Fourier Transform algorithm (PME-FFT). We used the AMBER FF19SB force field [4] for proteins, the GLYCAM_06j force field [5] for the glycan and TIP3P [6,7] for water.

Minimization was run for all systems with 5000 maximum cycles; the first 2500 cycles used the steepest descent algorithm. Positional restrains (1 kcal mol^{-1} angstrom^{-2}) were placed on all protein and glycan atoms (wt: 1-303, wtg: 1-308, del: 1-300, and delg: 1-305). Following minimization, the systems were equilibrated under the NVT ensemble, raising the temperature stepwise to 300K (50K, 100K, 200K, and 300K) with 125 ps per step, using the same positional restraints as during the minimization.

The production simulations were run under the NPT ensemble, with temperature control at 300K using Langevin dynamics (friction coefficient at 1ps^{-1}), potential energy control (nonbonded cutoff at 9 Å), and pressure control using Montecarlo Barostat (isotropic with target pressure at 1 bar). The SHAKE [8] algorithm constrained all protein hydrogen bonds, while water molecules were treated with SETTLE [9]. The systems were simulated stepwise for 3μs (100 ns per step). The energies, coordinates, and restart files were printed every 100 ps.

The minimization step was performed with the primary molecular dynamics engine pmemd implementation in AMBER 22 [10], while the equilibration and production runs were performed with single precision pmemd.cuda_SPFP in AMBER 22.

References:

[1] Jo S, Kim T, Iyer VG, Im W. CHARMM-GUI: A web-based graphical user interface for CHARMM. Journal of Computational Chemistry 2008;29:1859–65. https://doi.org/10.1002/jcc.20945.

[2] Brooks BR, Brooks III CL, Mackerell Jr. AD, Nilsson L, Petrella RJ, Roux B, et al. CHARMM: The biomolecular simulation program. Journal of Computational Chemistry 2009;30:1545–614. https://doi.org/10.1002/jcc.21287.

[3] Lee J, Hitzenberger M, Rieger M, Kern NR, Zacharias M, Im W. CHARMM-GUI supports the Amber force fields. The Journal of Chemical Physics 2020;153:035103. https://doi.org/10.1063/5.0012280.

[4] Tian C, Kasavajhala K, Belfon KAA, Raguette L, Huang H, Migues AN, et al. ff19SB: Amino-Acid-Specific Protein Backbone Parameters Trained against Quantum Mechanics Energy Surfaces in Solution. J Chem Theory Comput 2020;16:528–52. https://doi.org/10.1021/acs.jctc.9b00591.

[5] Kirschner KN, Yongye AB, Tschampel SM, González-Outeiriño J, Daniels CR, Foley BL, et al. GLYCAM06: A generalizable biomolecular force field. Carbohydrates. Journal of Computational Chemistry 2008;29:622–55. https://doi.org/10.1002/jcc.20820.

[6] Jorgensen WL, Chandrasekhar J, Madura JD, Impey RW, Klein ML. Comparison of simple potential functions for simulating liquid water. Journal of Chemical Physics 1983;79:926–35. https://doi.org/10.1063/1.445869.

[7] Neria E, Fischer S, Karplus M. Simulation of activation free energies in molecular systems. Journal of Chemical Physics 1996;105:1902–21. https://doi.org/10.1063/1.472061.

[8] Ryckaert J-P, Ciccotti G, Berendsen HJC. Numerical Integration of the Cartesian Equations of Motion of a System with Constraints: Molecular Dynamics of n-Alkanes. Journal of Computational Physics 1977;23:327–41. https://doi.org/10.1016/0021-9991(77)90098-5.

[9] Miyamoto S, Kollman PA. Settle: An analytical version of the SHAKE and RATTLE algorithm for rigid water models. Journal of Computational Chemistry 1992;13:952–62. https://doi.org/10.1002/jcc.540130805.

[10] Case D, Aktulga HM, Belfon K, Ben-Shalom I, Berryman J, Brozell S, et al. Amber 2022. 2022. https://doi.org/10.13140/RG.2.2.31337.77924.
