# Supplementary figures and images for: Assessing structure-function impacts on Vitellogenin by leveraging allelic variant found in honey bee subspecies Apis mellifera mellifera
Source: iScience. 2025 Jul 29;28(9):113241. doi: 10.1016/j.isci.2025.113241 (PMC12496180; doi:10.1016/j.isci.2025.113241)

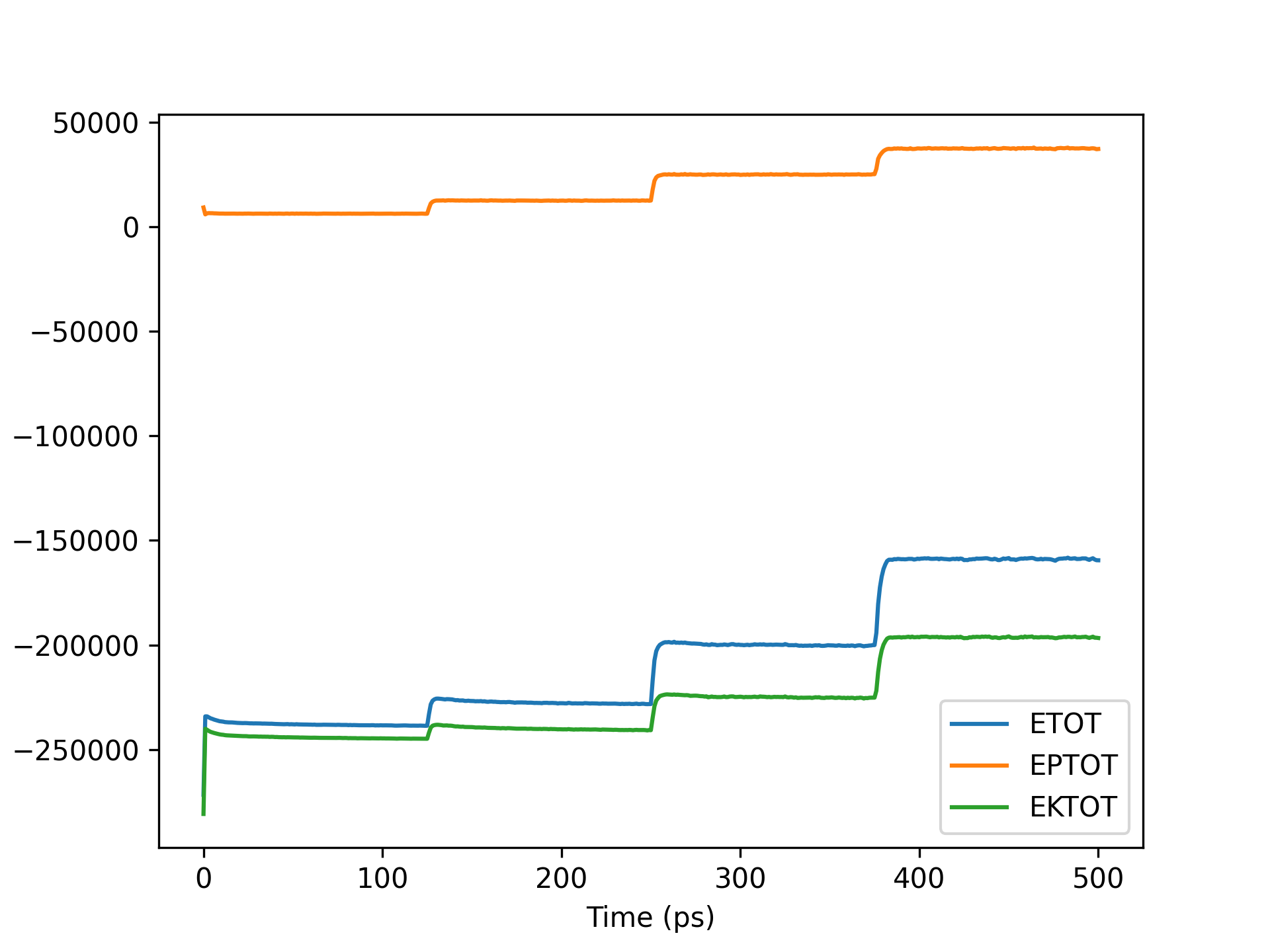

Supplement: Data S1. Data and code for molecular dynamics [file mmc2.zip › MD_analysis/inputfiles/del/Energy wt 0-300K.png]

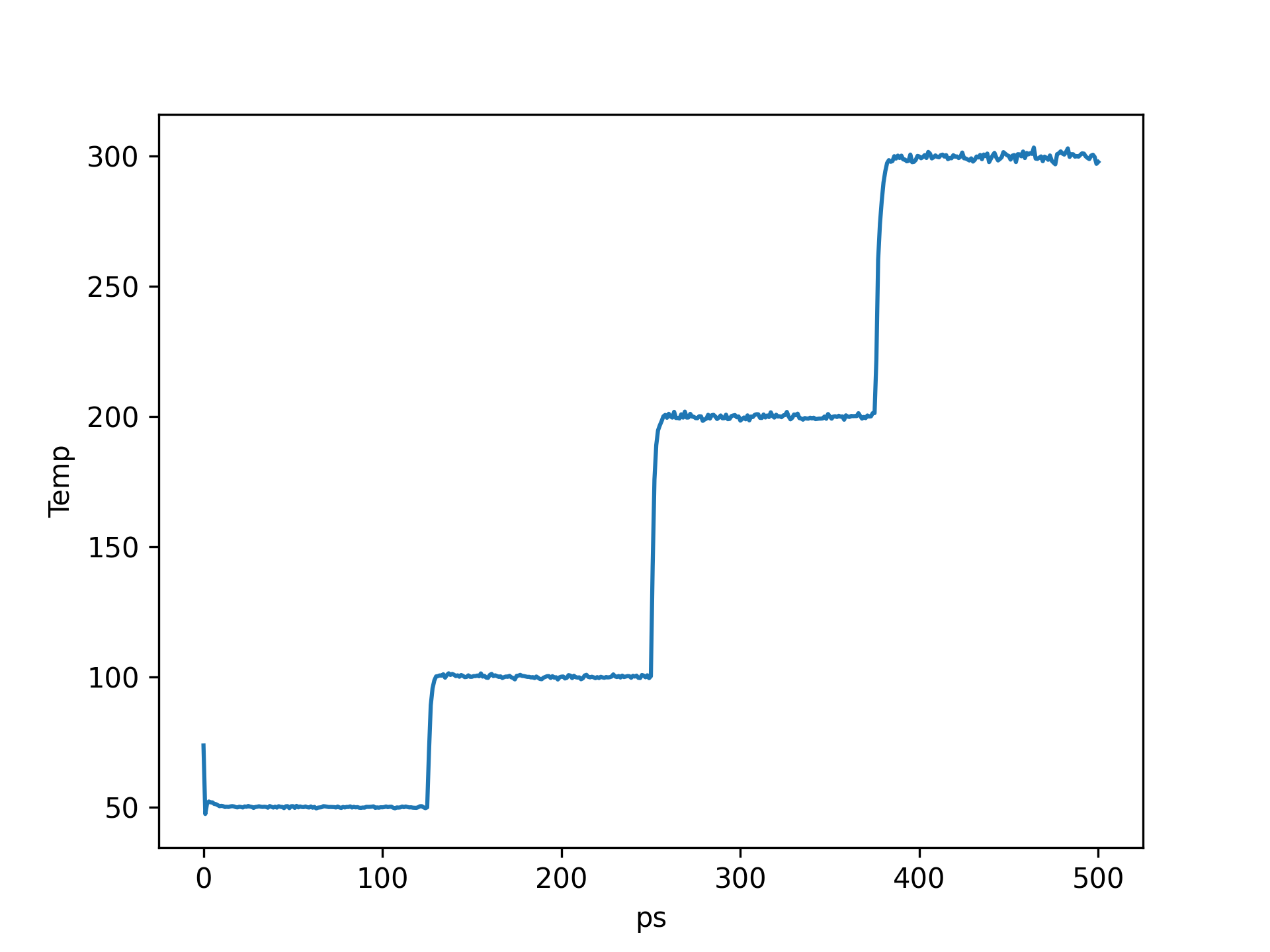

Supplement: Data S1. Data and code for molecular dynamics [file mmc2.zip › MD_analysis/inputfiles/del/Temp run wt 0-300K.png]

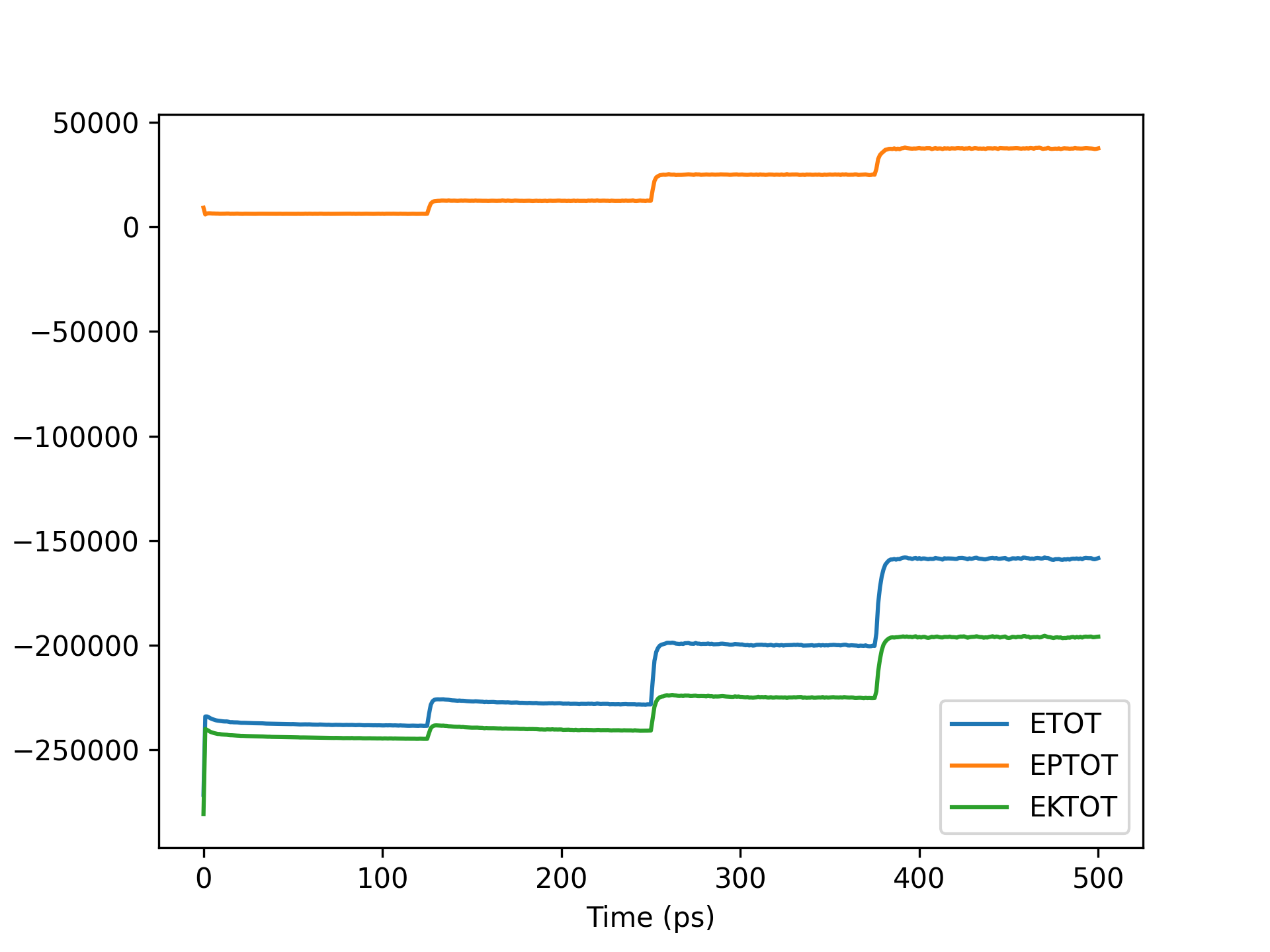

Supplement: Data S1. Data and code for molecular dynamics [file mmc2.zip › MD_analysis/inputfiles/delg/Energy wt 0-300K.png]

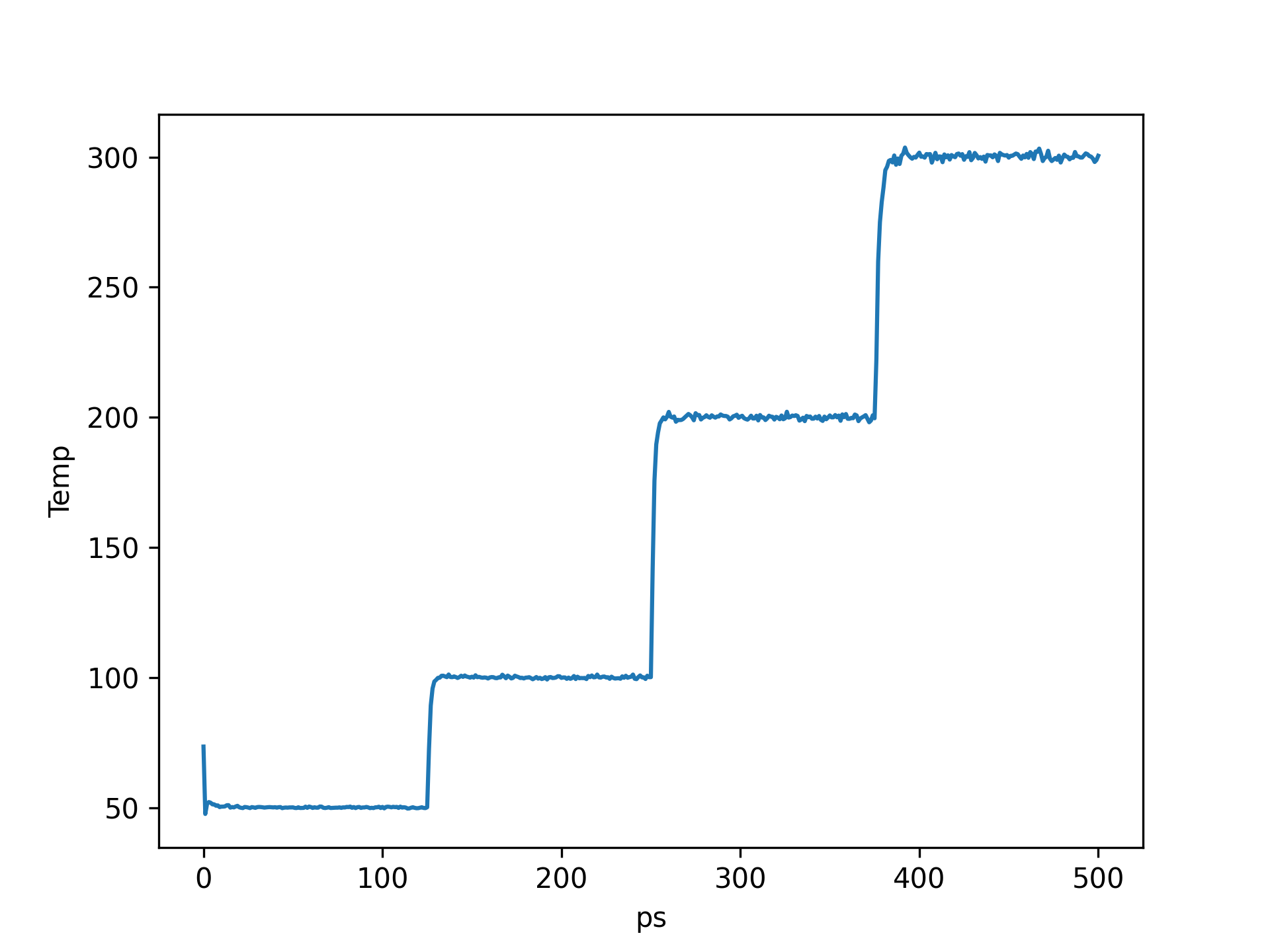

Supplement: Data S1. Data and code for molecular dynamics [file mmc2.zip › MD_analysis/inputfiles/delg/Temp run wt 0-300K.png]

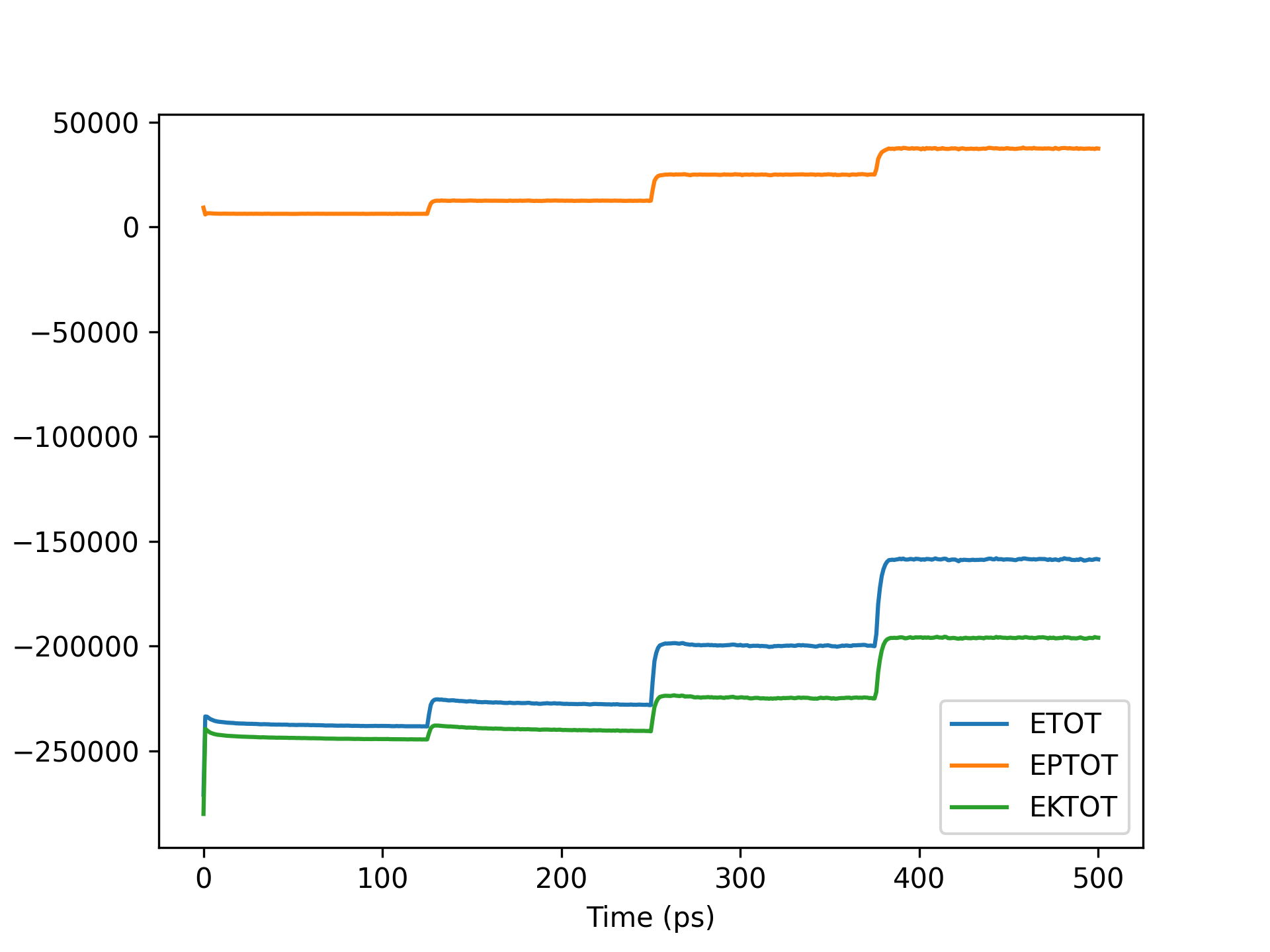

Supplement: Data S1. Data and code for molecular dynamics [file mmc2.zip › MD_analysis/inputfiles/wt/Energy wt 0-300K.png]

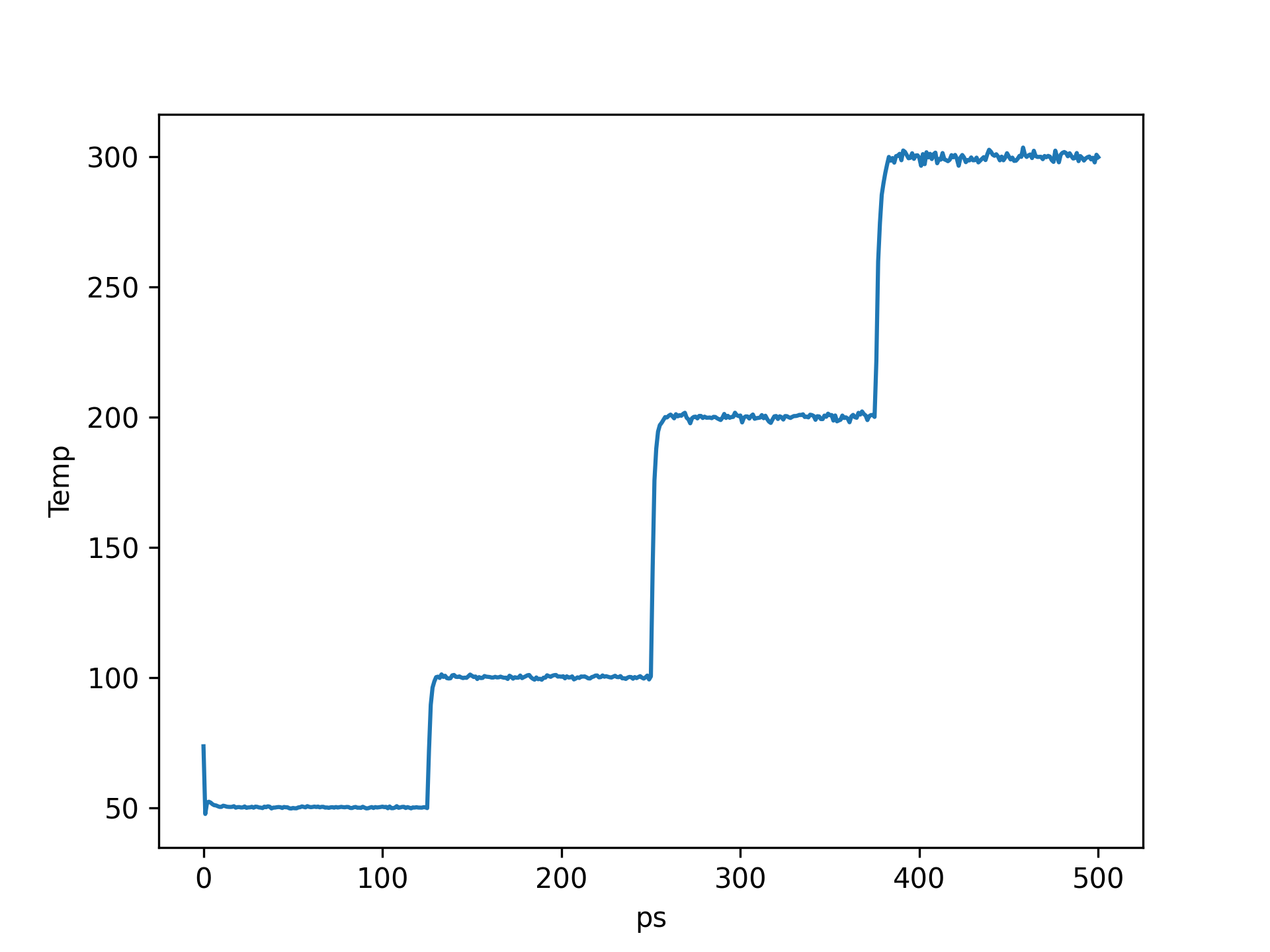

Supplement: Data S1. Data and code for molecular dynamics [file mmc2.zip › MD_analysis/inputfiles/wt/Temp run wt 0-300K.png]

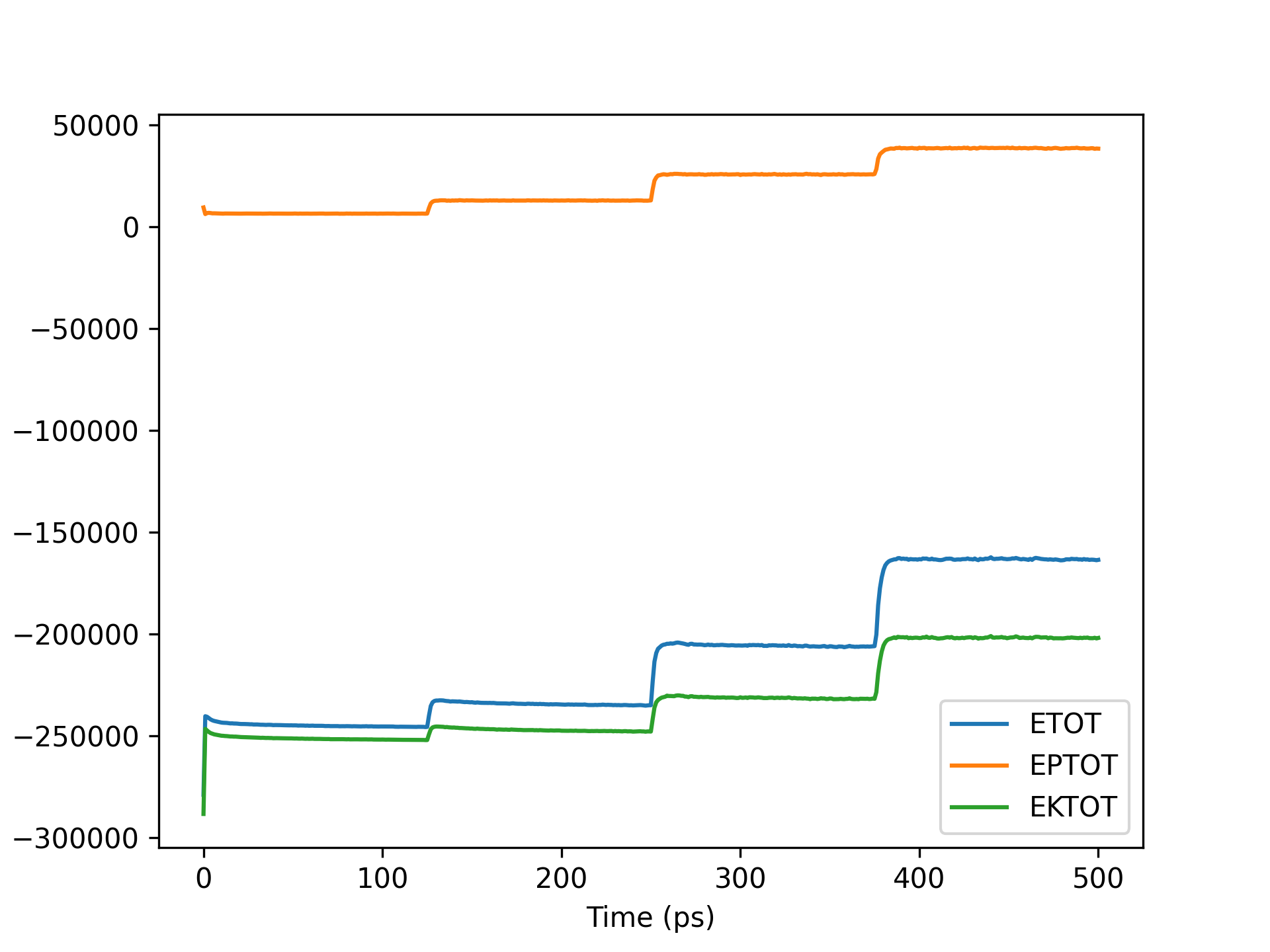

Supplement: Data S1. Data and code for molecular dynamics [file mmc2.zip › MD_analysis/inputfiles/wtg/Energy wt 0-300K.png]

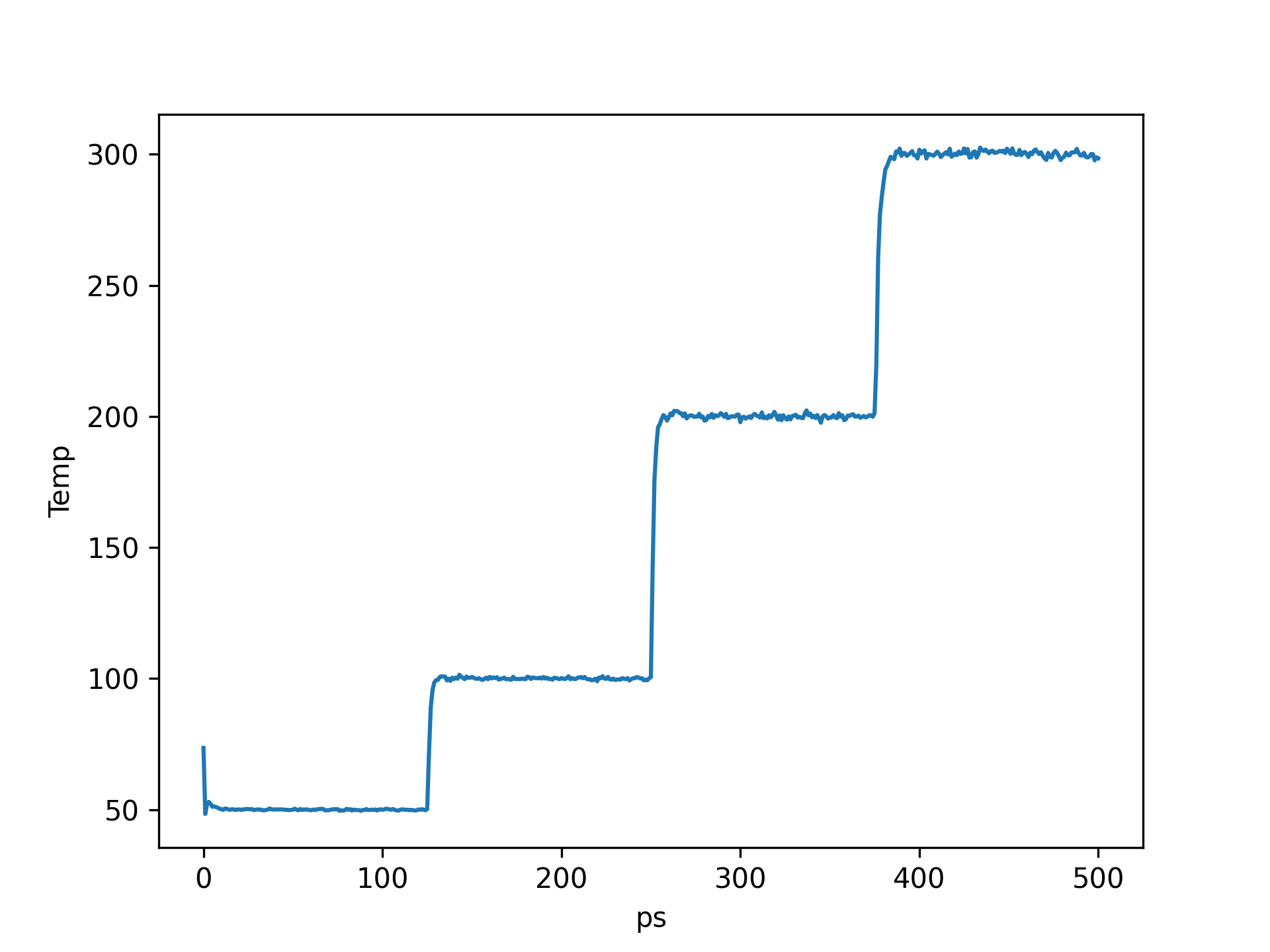

Supplement: Data S1. Data and code for molecular dynamics [file mmc2.zip › MD_analysis/inputfiles/wtg/Temp run wt 0-300K.png]
